# Speech-in-noise recognition as a predictor of academic achievement

**DOI:** 10.1371/journal.pone.0324998

**Published:** 2025-06-04

**Authors:** Dani Tomlin, Patrick Bowers, Benjamin Zonca, Kelley Graydon, Jocelyn Phillips, Gary Rance

**Affiliations:** 1 Department of Audiology and Speech Pathology, The University of Melbourne, Melbourne, Australia; 2 Faculty of Education, The University of Melbourne, Melbourne, Australia; Max-Planck-Institut fur Kognitions- und Neurowissenschaften, GERMANY

## Abstract

**Background:**

The capacity to listen effectively greatly influences learning. Speech understanding in noise, a crucial element of listening, involves the ability to comprehend and interpret speech despite surrounding auditory distractions or background noise. While numerous studies have examined the effect of noise on academic success, there is a noticeable lack of research focusing on how a student’s skill in understanding speech amidst noise connects to their unique educational achievements and overall learning journey. This study explores the relationship between listening ability and educational achievement.

**Design:**

108 primary school aged children; 55 from Grade 3 (mean age of 8 years 9 months) and 53 from Grade 5 (mean age of 10 years 7 months) participated in this study. Children completed both a listening skills assessment, using the Sound Scouts platform and the National Assessment Program - Literacy and Numeracy (NAPLAN). Interactions between results were explored.

**Results:**

A significant interaction between speech-in-noise ability and reading was seen in the combined cohort. When exploring interactions between variables within year levels, significant correlations between literacy (reading, grammar/punctuation, writing and spelling) outcomes, but not numeracy, were found in the Grade 3 children. No significant interaction between listening skills and academic achievement were observed in the Grade 5 cohort.

**Summary:**

The link between speech-in noise ability and literacy development provides insight into overlapping processes in both skills and their developmental trajectories. This highlights the importance of identifying not only the role that noise itself plays in learning, but the skills that support the ability to manage classroom listening environments. This research has implications for early hearing screening programs, teaching approaches, and interventions focused on enhancing the learning environment for all students.

## Introduction

For effective teaching and learning, successful transfer of knowledge from educator to student must occur. The student’s listening ability is a key factor in this exchange as it forms the basis for understanding and the processing of spoken information. The negative impact of hearing loss on academic achievement has been comprehensively shown in the literature [1–3], however less is known about how listening ability in children without diagnosed hearing impairment can influence academic outcomes. Listening is an active skill that plays a pivotal role in learning. It encompasses the ability to decode, interpret, and synthesize auditory information, making it a key component of academic success [4]. Listening is an interactive process that allows meaning to be drawn from the sounds heard [5], and can be measured by subjectively evaluating skills such as following instruction, understanding speech and maintaining concentration in noise. Real-life listening ability may quantify how well a student is able to listen in the environments they normally experience. In a primary school classroom, effective listening is not limited to the comprehension of spoken language alone. It extends to interactions with classmates, discussions, and group activities – all of which are integral to learning. Whilst more than half of classroom learning consists of active listening activities [6], the classroom environment often presents significant challenges to listening in the form of ambient, background noise and distractions, which can impede a child’s ability to engage with and absorb educational content [7–10].

In educational settings, ambient noise is an ongoing challenge, whether it be the voices of fellow children, the hum of electronic devices, or external environmental sounds. These auditory distractions can significantly affect children’s speech perception, making it difficult for them to fully grasp the content being delivered by teachers or peers. As the intensity of the background noise increases, the signal to noise ratio (i.e., the level of the speaker’s voice relative to the noise [SNR]) decreases, resulting in a degradation of speech intelligibility. For this reason, SNR is the focus of most evaluations of classroom acoustics [11–13]. A recent review conducted by Minelli and colleagues (2022), established that when noise levels exceed acceptable levels and poor SNR result (≤+5 decibels [dB]), children below the age of 12 are unable to achieve satisfactory academic performance. The type of noise, in addition to the overall noise level, has also been recognized as crucial [14]. Notably, distracting speech noise has been shown to have an adverse effect on academic performance, as highlighted in a review of the effect of noise on cognitive performance in children [10]. For instance, meaningful speech has been shown to result in greater impairments on verbal tasks than meaningless speech [10]. Meaningful speech may engage semantic functions that compete with ongoing academic literacy tasks, and when trying to ignore a competing signal of meaningful speech, such as the child’s name, there is an increased burden of attention. A phenomenon, known as the Irrelevant Sound Effect (ISE), is outlined in Puglisi et al.’s study on reduced reading speed in 7–8-year-old children in poor acoustic environments [15]. The irrelevant sound effect describes the reduced performance of adults and children on visual tasks such as memorisation of items in the presence of background speech noise, even if this speech is not relevant to the task being performed [16–18].

Auditory factors such as increased listening effort alongside masking effects have been proposed as mechanisms for disruption of learning. Rance et al. [19] suggest a causal link between auditory factors and cognitive effects, implicating attention and cognitive resource allocation (CRA) resulting in poor academic progress due to excessive noise levels for some children. They found that classrooms with better acoustics had higher rate of improvement in reading fluency across a school term and this effect was heightened for students with poor speech in noise skills. However, quantifying the ‘link’ between listening effort and academic progress remains challenging due to a lack of direct measures of listening effort [19].

To better understand how noise and learning interact, more clarity on not only how (but which) learning outcomes are affected by children’s ability to manage background noise is needed. Is this seen across the breadth of learning or limited to a particular domain? Noise in the classroom environment has been reported to disrupt a wide range of educational processes as noise can impede a child’s ability to concentrate and maintain attention, resulting in reduced comprehension of instructional material [20]. Klatte and Lachmann [21] have shown that excessive noise levels can hinder students’ working memory, making it challenging for them to retain and manipulate information, which is integral to tasks utilising problem-solving and critical thinking. The ability to engage in effective classroom discussions and participate in group activities can be compromised in noisy environments, potentially impacting social and communication skills development. Thus, it is evident that noise has the potential to affect a spectrum of learning outcomes.

While noise may disrupt all learning domains, the interference in reading learning may relate to comprehension and decoding skills. For instance, studies such as those by Shield and Dockrell [20] have shown that background noise can hinder the ability to understand and process written text, leading to reduced reading fluency and comprehension. This disruption in reading arises from the need for effective phonological processing and semantic integration, both of which can be impeded by noise. A review by Mealings [22] concluded that noise exposure has an adverse effect on literacy performance in children. In contrast, another review by Mealings [23] found varied impacts of noise on children’s numeracy outcomes. The effect of noise on numeracy learning may centre on working memory and problem-solving tasks. Research like that of Klatte and Hellbrüuck [24] indicates that noise in educational settings can challenge children’s working memory, making it more difficult for them to perform mental calculations and engage in problem-solving tasks. This suggests that while noise may affect both reading and numeracy learning, the cognitive processes involved in each domain may respond differently to noise interference.

While various studies have explored the impact of noise on academic performance, there is a distinct scarcity of research that specifically investigates how an individual’s ability to perceive speech in noisy environments correlates with their distinct educational achievements and overall learning experience. Rance, Dowell, & Tomlin [19] demonstrated that noisy classroom environments impact on learning and linked lower levels of speech-perception in noise with lower levels of reading fluency development in primary school aged children. Additionally, researchers have identified lower levels of speech-in-noise recognition as one characterizing feature of dyslexia [25,26]. This research gives cause to further explore the relationship between speech in noise recognition and learning.

The primary objective of this study was to investigate the direct link between speech-in-noise ability and learning outcomes in educational settings. We aimed to determine whether variations in speech in noise proficiency are associated with differences in academic performance across the breadth of learning, or if certain domains are more impacted than others. The findings of this study hold the potential to inform educational practices, teaching strategies, and interventions aimed at optimizing the learning environment for all students.

## Methods

### Participants

A total of 108 children from 7 government primary schools across the state of Victoria in Australia were included in this study. Mean age of all participants was 9 years 8 months, with a range of 8 years 0 months to 11 years 6 months. Our sample included 55 children from Victorian year level 3 and 53 in year 5. Mean age of Grade 3 children was 8 years 9 months (Range 8 years 0 months to 9 years 8 months), while mean age of Grade 5 children was 10 years 7 months (Range 9 years 9 months to 11 years 6 months). Decile scores for the relative socioeconomic advantage and disadvantage, calculated using the postcodes of the included schools were collected. A score of 1 represented the lowest 10% of scores in Australia and scores of 10 represented the highest 10% [27]. The locations of two schools were in areas scoring a 1, three school locations scored a 2, one school a 3 and one school a 5 [27]. Children were invited to participate if they were in Victorian Grades 3 and 5, as the National Assessment Program - Literacy and Numeracy (NAPLAN) occurs for all children annually in these year levels [28].

This study was approved by the University of Melbourne Human Research Ethics Committee (193825.1). Informed written consent from caregivers and verbal assent from each child was obtained for all participants.

### Hearing assessment

Hearing assessments occurred between May and September 2022. Each child completed a hearing screening using the automated hearing assessment software, Sound Scouts [29]. Children completed the testing on iPads with Sennheiser HD300 headphones, under the supervision of an Audiologist. Assessments occurred in quiet, unoccupied rooms within school grounds (such as empty classrooms or office spaces). Each assessment ran for approximately 10 minutes.

Sound Scouts is presented as a game on tablet devices, with interactive multimedia and graphics intended to engage and interest children [29]. The game involves three different assessments of a child’s hearing and listening ability, presented sequentially. Each test is introduced verbally, with instructions audible through the headphones. The first test of the game is a speech-in-quiet test. During this test, target words are presented aurally to the child, one ear at a time. This test results in a speech recognition in quiet threshold, for each ear, and results are used to calibrate the volume required for the subsequent assessments. Following the speech-in-quiet test is a tone-in-noise test, which tests both ears and results in another individual score. Finally, a speech-in-noise test proceeds until a speech reception threshold in noise is established, representing the average of SNRs presented over the test period. Speech-in-quiet and tone-in-noise assessments aim to identify children with varying patterns of hearing loss, whereas the measure of speech-in-noise additionally aims to identify children with auditory processing deficits [29]. For further detail about each sub-test, the original article by Dillon et al. [29], may be consulted.

Each of the tests described is assessed for validity and scores are given for performance on each of the three tests. This score represents the child’s performance; calculated by comparing the result to normative data, to give a score that considers a child’s performance in relation to typically developing children of their age, as described by Dillon and Mee [29]. Scores are then translated into pass-fail results, and are summarised as: fail (0–70), borderline (71–78) and pass (79+). Despite Sound Scouts being a screening tool rather than diagnostic test, the test has been shown to accurately identify abnormal auditory function, with a sensitivity of 85% to correctly identify any hearing loss in children increasing to 100% to identify an average hearing loss of worse than 30dBHL [29]. Specificity of between 95–98% has also been demonstrated, indicating a low rate of false refers [29,30].

### Academic achievement measure

Academic achievement measures in the form of standardised scores from the NAPLAN were obtained following consent from parents. Each child’s most recent NAPLAN results were obtained (their May 2022 assessment). Five academic domains are assessed through NAPLAN: Reading, Numeracy (including algebra, geometry, measurement and statistics and probability), Spelling, Writing, and Grammar & Punctuation. Raw scores for each test represent questions answered correctly and are converted to normalized scale scores [28]. These scales range from approximately 0 (lowest performance) to 1000 (highest performance) and show student accomplishment in relation to national minimum standards for learning.

### Statistical analyses

Descriptive statistics of our sample were generated, as were Pearson correlation coefficients for all pairs of Sound Scouts test scores and NAPLAN domains. Mixed effects regression models were then run to investigate associations between all academic measure scale scores and Sound Scouts scores for children depending on their year level. School was treated as a random effect, year level as a categorical fixed effect and three Sound Scouts listening measures were considered as continuous predictors: speech-in-quiet score (2^nd^ ear), tone-in noise score and speech-in-noise score. Speech-in-quiet score (1^st^ ear) was not included as a predictor as it was found to be highly correlated with speech-in-quiet score (2^nd^ ear). Statistical analyses were performed using Minitab (version 21.3) and R (version 4.4.1) software.

Standard diagnostic plots were checked for each regression model. Collinearity among predictor variables was evaluated via the variance inflation factor (VIF), a measure of the impact of multicollinearity on the fitted regression model. All predictors for all of the models had a VIF of less than 2; with values below 3 demonstrating tolerable levels of collinearity for the purpose of regression analysis [31,32].

The linear mixed model results are presented as standardised effect sizes. For the dichotomous predictor (year level) the standarised effect size was calculated as the estimated difference in means between year levels, divided by the residual standard deviation from the mixed effect model. The residual standard deviation is the standard deviation of errors from the fitted model (after accounting for the predictor variables, including differences in mean between schools, which is treated as a random effect). For continuous predictors and interaction terms with year level, the standarised effect size was calculated by refitting the model but normalizing the response and all predictor variables [33].

## Results

### Hearing assessment

**Fig 1:**
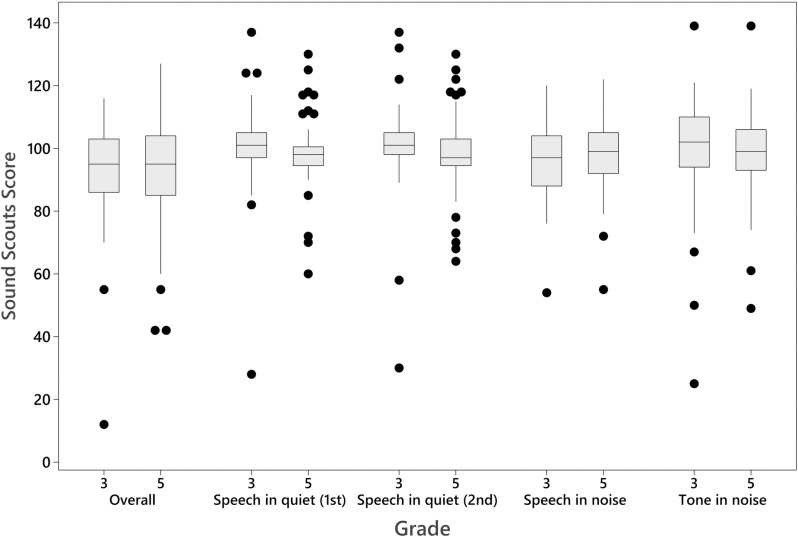
Sound scouts scores by academic year level and hearing assessment sub-test. Scores for overall Sound Scouts score, first ear speech-in-quiet, second ear speech-in-quiet, speech-in-noise and tone-in-noise are reported. Score cutoffs: fail (0-70), borderline (71-78), pass (79+).

All children completed both the hearing assessment (Sound Scouts) and NAPLAN within 4 months of one another. Scaled hearing assessment scores for Grades 3 and 5 are presented in Fig 1.

### Academic achievement

**Fig 2:**
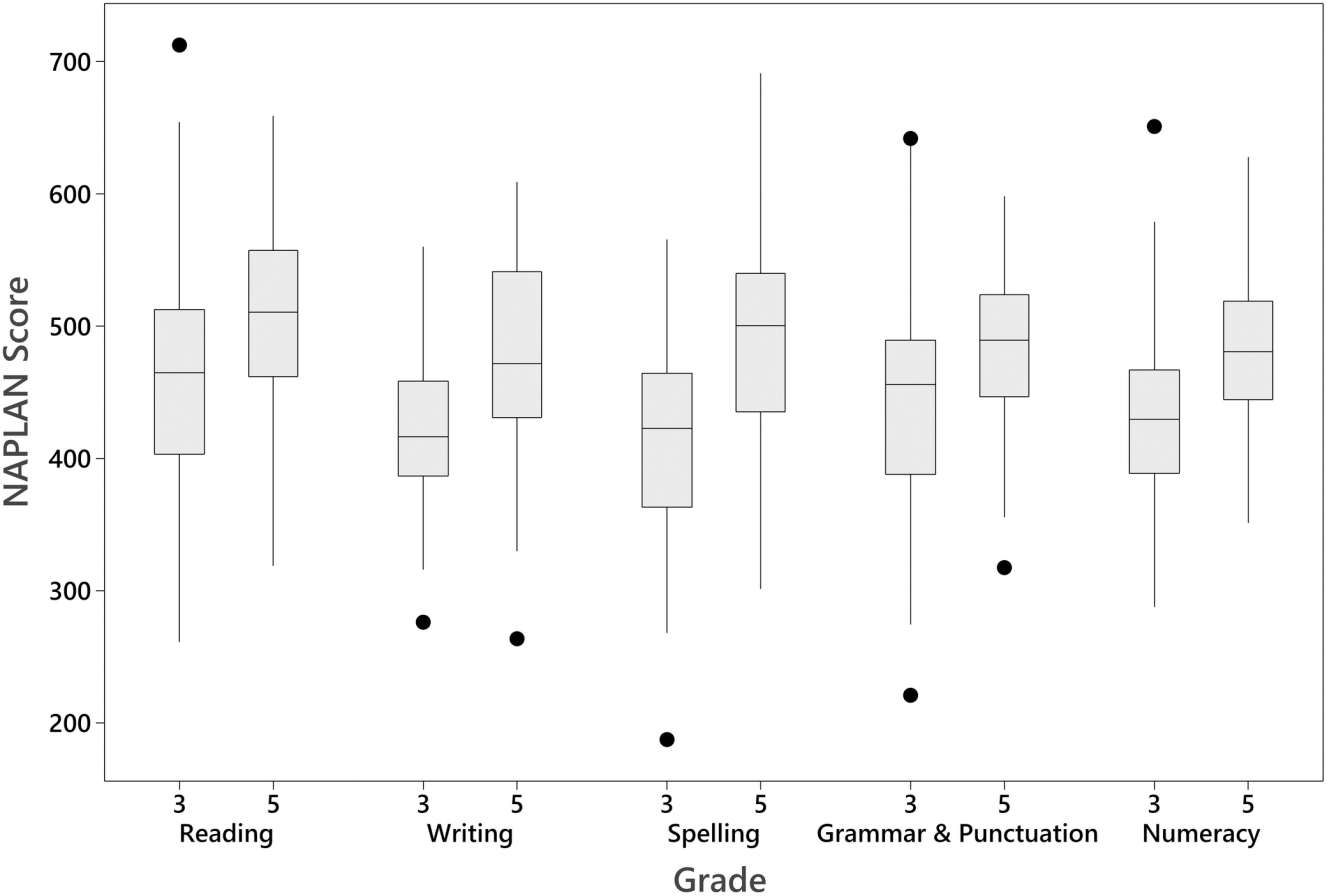
NAPLAN scores for each of the 5 academic measures, by year level.

Academic performance scores from NAPLAN had a group mean of 462.7 (90.4 SD) for Reading, 432.2 (74.3SD) for Numeracy, 432.3 (95.4SD) for Spelling, 438.3 (69.7 SD) for Writing, and 449.0 (89.9 SD) for Grammar and Punctuation. NAPLAN scores are reported on a common scale across all year levels, allowing comparisons of performance for students over time. Mean scores were higher across all measures for Grade 5 compared to Grade 3, as is expected from national mean scores. Grade 3 academic performance scores from NAPLAN had a group mean of 460.3 (94.2 SD) for Reading, 429.3 (69.4SD) for Numeracy, 413.0 (83.4SD) for Spelling, 423.6 (54.9 SD) for Writing, and 451.1 (89.0 SD) for Grammar and Punctuation. Grade 5 academic performance scores from NAPLAN had a group mean of 503.1 (76.0 SD) for Reading, 483.87 (56.8SD) for Numeracy, 486.6 (87.3SD) for Spelling, 479.6 (74.2 SD) for Writing, and 482.6 (69.2 SD) for Grammar and Punctuation. A representation of scores by each year level can be seen in Fig 2. NAPLAN data was also explored to examine the variance by year level. Levene tests of equal variance showed no significant difference across all NAPLAN domains of Reading, Writing, Spelling, Grammar/Punctuation and Numeracy (*p *= .19;.89;.11;.26 &.53 respectively).

### Associations between measures

Table 1 describes correlation between hearing assessment and NAPLAN scores. Strong relationships between hearing assessment subtests were found, with all being significantly correlated with one another. This was also the case for NAPLAN scores. A statistically significant correlation between reading scores and speech-in-noise (*p* = .020) as well as overall Sound Scouts score (*p *= .004) was found.

**Table 1:** Correlation coefficients for all children **p < .01 *p < .05.

	Overall Sound Scout Score	Tone in Noise	Speech in Noise	Speech in quiet (1^st^ ear)	Speech in quiet (2^nd^ ear)	Reading	Spelling	Writing	Grammar & Punctuation
**Tone in noise**	0.70**								
**Speech in noise**	0.78**	0.30**							
**Speech in quiet (1**^**st**^ **ear)**	0.56**	0.31**	0.40**						
**Speech in quiet (2**^**nd**^ **ear)**	0.67**	0.32**	0.48**	0.70**					
**Reading**	0.23**	0.10	0.19*	0.08	0.15				
**Spelling**	0.13	0.02	0.11	0.00	0.13	0.76**			
**Writing**	0.12	0.00	0.07	0.00	0.14	0.67**	0.81**		
**Grammar & Punctuation**	0.13	−0.03	0.14	0.02	0.11	0.79**	0.76**	0.68**	
**Numeracy**	0.02	−0.04	0.06	−0.04	−0.02	0.64**	0.62**	0.53**	0.65**

### Mixed effects regression modelling for NAPLAN scores

Mixed effects regression models were fitted to predict NAPLAN scores. The mixed effects models included both fixed and random effects predictors. The fixed effects were year level (treated as a categorical predictor with two levels), speech perception in quiet score, tone perception in noise score, and speech perception in noise score. School was included as a random effect in each model. Separate models were created for each NAPLAN domain (reading, numeracy, spelling, writing, grammar/ punctuation). As noted previously, given the high and significant correlation between 1^st^ and 2^nd^ ear speech-in-quiet scores (coefficient 0.70; p < .001), only the second ear was included in the analysis.

The initial model (model 1) includes interactions between year level and each Sound Scouts outcome measure. Year level was a highly significant predictor of performance across all NAPLAN domains, with Year 5 students scoring better than Year 3 (Table 2). Speech-in-noise performance showed a significant interaction with multiple NAPLAN metrics (reading, grammar/punctuation and writing), and this interaction term showed a marginal effect for spelling. Furthermore, the standardized effect sizes for this interaction were consistently the second highest (after year level) among the predictors included in Model 1; these were also consistent in the direction of the association across all of the NAPLAN outcomes. Of the other Sound Scouts scores, only speech-in-quiet showed a significant (positive) association with a single NAPLAN metric (Spelling), however as noted the effect size was less than the year-level by speech-in-noise score.

**Table 2:** Model 1 of variables explaining NAPLAN outcomes, reporting standarised effect sizes, the 95% CI of the standardised effect size (in parentheses), and significance levels for the main effects, as well as their interactions with year level. Asterisks indicate statistically significant effects at different p-value thresholds (*p < 0.05, **p < 0.01, ***p < 0.001).

Effect	Reading	Grammar/ punctuation	Writing	Spelling	Numeracy
Year level	0.553 (CI: 0.039 to 1.066; p = 0.013*)	0.527 (CI: 0.008 to 1.046; p = 0.018*)	1.209 (CI: 0.683 to 1.736; p < 0.001***)	1.125 (CI: 0.607 to 1.642; p < 0.001***)	0.878 (CI: 0.375 to 1.382; p < 0.001***)
Speech in quiet	0.115 (CI: −0.175 to 0.405; p = 0.43)	0.028 (CI: −0.243 to 0.299; p = 0.84)	0.371 (CI: 0.099 to 0.642; p = 0.0082**)	0.219 (CI: −0.050 to 0.489; p = 0.11)	0.010 (CI: −0.300 to 0.321; p = 0.95)
Tone in noise	−0.046 (CI: −0.362 to 0.270; p = 0.77)	−0.145 (CI: −0.440 to 0.150; p = 0.33)	−0.087 (CI: −0.374 to 0.199; p = 0.54)	−0.156 (CI: −0.449 to 0.138; p = 0.29)	−0.023 (CI: −0.355 to 0.308; p = 0.89)
Speech in noise	−0.108 (CI: −0.390 to 0.173; p = 0.45)	−0.106 (CI: −0.369 to 0.157; p = 0.43)	−0.233 (CI: −0.494 to 0.028; p = 0.079)	−0.123 (CI: −0.385 to 0.139; p = 0.35)	0.048 (CI: −0.254 to 0.350; p = 0.75)
Year level * speech in quiet	0.066 (CI: −0.357 to 0.489; p = 0.76)	0.226 (CI: −0.170 to 0.623; p = 0.26)	−0.230 (CI: −0.631 to 0.172; p = 0.26)	−0.069 (CI: −0.464 to 0.325; p = 0.73)	−0.177 (CI: −0.619 to 0.266; p = 0.43)
Year level * tone in noise	0.006 (CI: −0.403 to 0.414; p = 0.98)	−0.124 (CI: −0.507 to 0.258; p = 0.52)	−0.091 (CI: −0.472 to 0.289; p = 0.63)	0.023 (CI: −0.358 to 0.403; p = 0.91)	−0.069 (CI: −0.494 to 0.355; p = 0.75)
Year level * speech in noise	0.483 (CI: 0.072 to 0.895; p = 0.022*)	0.614 (CI: 0.230 to 0.999; p = 0.0021**)	0.490 (CI: 0.092 to 0.889; p = 0.017*)	0.367 (CI: −0.016 to 0.750; p = 0.06)	0.209 (CI: −0.225 to 0.642; p = 0.34)

**Fig 3:**
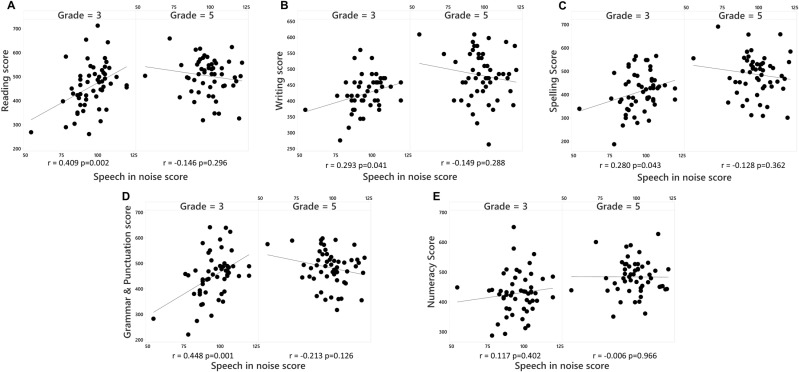
Scatterplots of Speech-in-noise scores for each year level (in panels) with A. Reading B. Writing C. Spelling D. Grammar & Punctuation and E. Numeracy.

The relationship of the two variables highlighted in the by examination of the standarised effect sizes for Model 1 (speech-in-noise and year-level) with the NAPLAN scores is shown in Fig 3. These results demonstrate the significant correlations between NAPLAN literacy measures and speech-in-noise ability in the Grade 3 cohort. No such correlation was seen in the Grade 5 cohorts. No correlation was observed in either year for numeracy performance.

The results of model 1 suggest that speech-in-noise perception showed the most consistent interactions with year level, when predicting NAPLAN performance across domains. The speech-in-noise Sound Scouts measure is also most representative of students’ classroom listening environment. To focus on this effect, model 2 was created to investigate the impact of speech in noise ability, and the interaction of speech and noise and year level on NAPLAN outcomes, without the effects of speech-in-quiet and tone-in-noise results. Model 2 (Table 3) demonstrates that speech perception in noise ability showed effects for reading, grammar/punctuation, writing and spelling, however all of these relationships differed by year level. The sign of these effects was consistent with the correlation analysis as shown in Fig 3. In contrast to the relationship between speech-in-noise performance and literacy outcomes, speech-in-noise ability, nor its interaction with year level were significant predictors of numeracy scores.

**Table 3:** Model 2, final model of variables explaining NAPLAN outcomes. As with Table 2, this is reported in terms of standarised effect sizes, the corresponding CI, and significance levels for predictor variables. The standarised slope for Grade 3 is obtained by adding the “Speech in noise” and “Year level * speech in noise” coefficients, while the corresponding slope for Grade 5 can be read directly from the “Speech in noise” row. Asterisks indicate statistically significant effects at different p-value thresholds (*p < 0.05, **p < 0.01, ***p < 0.001).

Effect	Reading	Grammar/ punctuation	Writing	Spelling	Numeracy
Year level	0.509 (CI: 0.007 to 1.010; p = 0.019*)	0.473 (CI: −0.030 to 0.976; p = 0.03*)	1.099 (CI: 0.585 to 1.613; p < 0.001***)	1.065 (CI: 0.560 to 1.570; p < 0.001***)	0.927 (CI: 0.433 to 1.422; p < 0.001***)
Speech in noise	−0.090 (CI: −0.333 to 0.153; p = 0.46)	−0.163 (CI: −0.398 to 0.072; p = 0.17)	−0.131 (CI: −0.367 to 0.104; p = 0.27)	−0.098 (CI: −0.329 to 0.132; p = 0.4)	0.053 (CI: −0.202 to 0.308; p = 0.68)
Year level * speech in noise	0.543 (CI: 0.190 to 0.897; p = 0.003**)	0.721 (CI: 0.380 to 1.062; p < 0.001***)	0.395 (CI: 0.045 to 0.745; p = 0.027*)	0.378 (CI: 0.043 to 0.712; p = 0.027*)	0.093 (CI: −0.277 to 0.462; p = 0.62)

## Discussion

The results of this study showed an association between children’s ability to follow speech in noise and literacy performance. Importantly, this result was only seen in the younger (Grade 3) cohort, highlighting a changing relationship over time across age and development. The identified association was also not evident in the numeracy domain across either year level. The association between younger children’s speech-in noise and literacy performance aligns with previous results showing that children with speech-in noise difficulties are more impacted by noisier classrooms when measuring reading development [19]. The correlation between speech in noise ability and literacy in younger children, but not numeracy development also suggests that the association between speech in noise ability and literacy development is more complex than solely a disruption of information transfer.

### Linking speech-in-noise perception to academic development

There are many possible factors that may contribute to the link between literacy and speech-in-noise performance in younger children including auditory, cognitive and linguistic factors. Given an association was only observed between speech-in-noise and literacy performance, and not numeracy performance, and the relationship was only seen in younger children, this may give insight into the possible mechanism driving the association. Children with a hearing loss have been shown to have poorer speech-in-noise ability than normally hearing children [34,35]. It is however expected that rates of hearing impairment would be similar between Grade 3 and Grade 5 students, and only a very small number of children in the present study received a fail result on the speech-in-quiet Sound Scouts measure, aimed at identifying children with any hearing loss [29]. Given the identified change in relationship between speech-in-noise and academic performance over time, with an association only identified in younger children, it is unlikely that this can be explained by hearing changes.

Cognitive factors, including working memory, attention and executive functions can all impact children’s performance on measures of speech-in-noise recognition [36–38]. While development of cognitive factors throughout childhood may explain some of the identified relationship between speech-in-noise and academic performance in younger children, the influence of cognitive factors would be expected to impact both numeracy and literacy performance. Given the identified association was only present for literacy and not numeracy measures, we therefore postulate that other mechanisms are likely driving the link between literacy performance and speech-in-noise ability in younger children identified in the present study.

Language ability also influences speech-in-noise recognition ability in children, particularly for complex stimuli [37,38]. There are three phonological processes associated with the development of knowledge of the sounds of language and written language: phonological awareness, phonological memory, and phonological recoding [39]. Phonological awareness is the ability to identify and manipulate sounds (e.g., the ability to replace the phoneme ‘c’ in the world ‘cat’ with the phoneme ‘m’). Phonological memory refers to the ability to store sound information in the short-term memory (e.g., the ability to repeat strings of phoneme information). Phonological recoding is the ability to retrieve phoneme information from the long-term memory. For children with typical hearing each process is understood as a predictor of literacy ability, with phonological awareness and phonological recoding (and less so phonological memory) linked to spelling ability [40]. While there are likely multiple factors contributing to the identified link between literacy and speech-in-noise performance in younger children, a proposed contribution is through their relationship to the development of phonological processing.

### Phonological awareness, speech-in-noise perception and literacy development

The potential link between lower proficiency in speech-in-noise perception and assessed literacy skills in Grade 3 students may stem from a disruption in a student’s early development of phonological awareness (PA). Phonological awareness specifically as a predictor of literacy ability is widely discussed and acknowledged as a critical component for early literacy development, necessitating the integration of speech, language, and auditory processing [41–43]. PA is described as the capacity to identify and manipulate sounds in spoken language without relying on written text [44,45] and encompasses larger elements such as syllables, onsets and rhymes, and smaller phonemes [46]. Research has indicated that amplitude rise time and frequency discrimination of speech sounds impact PA development in younger readers [47]. Younger readers with higher levels of PA consistently demonstrate higher performance on text-based assessments of reading and spelling [48].

The strong correlation between speech-in-noise and text-based reading achievement found in our data supports the suggestion that speech-in-noise may correlate to PA, and PA correlates to word-level reading ability. This is supported by the work of Boets and colleagues [49] who concluded that whilst there is an overrepresentation of speech-in-noise deficits in children with literacy delays, this is not a causal relationship, but a result of the influence of PA in both tasks. In contrast, Vandewalle and Boets [50] found an association between speech perception ability and literacy performance beyond phonological processing skills. Despite the significant association between speech-in-noise performance and literacy skills, they explain only a small amount of the variance in literacy outcomes. Additional factors this research has not explored, such as language and vocabulary, working memory or attention ability may be influencing the association between speech-in-noise and literacy performance.

Our data shows no correlation between speech-in-noise perception and numeracy. This finding follows the limited evidence base relating speech-in-noise perception deficits [51]or PA deficits [52] to decreased numeracy performance. In both cases, speech-in-noise and PA deficits may affect basic skills of computational fluency and rapid naming (particularly when questions are presented verbally) with no clear relationship for more complex areas [53]. Furthermore, any significant correlation between PA and mathematics tends to occur in the earliest years of schooling, when tasks are typically focused on counting and number identification [52]. One study of NAPLAN Numeracy achievement of government school students in Victoria showed that reading comprehension had a mediating effect only on problem-solving ability and not mathematics achievement [54]. Given that speech-in-noise perception has a significant predictive relationship with PA, the lack of association between lower speech-in-noise perception and mathematics performance in this study may be explained by lower reading demands of NAPLAN Numeracy questions [55]. Further research is needed to better understand the relationship between speech-in-noise perception and tested numeracy performance.

### Effect only seen in younger children

Although there was a lack of a main effect of speech-in-noise on academic performance, when the interaction of year level was included in the mixed effects model, highly significant associations were observed. The lack of association between speech-in-noise perception and tested reading ability in the Grade 5 cohort, compared to the significant association seen in the Grade 3 cohort, raises questions about speech-in-noise and PA relationships over time. Heidari et al. [56] suggest that speech-in-noise perception improves linearly over time regardless of starting ability, and this trend remains for PA, where PA skills measured in the early years strongly correlate to PA skills at 17-years [57]. One study discusses the trajectory of phonological awareness between Grade 3 and 5 in students with existing reading and/or spelling learning difficulties [58]. The authors identified a persistent deficit in phonological awareness throughout the primary years. Speech perception in noise ability is underpinned by linguistic, auditory processing and cognitive factors [59] and may follow a different developmental trajectory to PA. The ability of children to rapidly access stored phonological knowledge to reconstruct degraded or masked speech is weaker compared to the adult listener and similarly the bank of stored representations of whole words is far smaller in children [25]. Adults are also better able to utilise contextual cues when reconstructing noise-obscured words within a sentence [60]. Cognitive factors such as attention are also associated with speech-in-noise ability and demonstrated to develop through childhood [61]. The most significant period of development occurs between 8 and 9 years of age [62], as demonstrated by Rebok et al. [63] in a longitudinal study. The authors showed significant changes in sustained attention ability occurred between 8 and 10 years of age, with only minor improvements beyond 10 years of age. As children age and speech-in-noise ability matures, the contribution, interaction between, and reliance on linguistic, cognitive and auditory factors will alter, whilst the trajectory of PA remains linear. This aligns with a reduced association between speech-in-noise and literacy ability in older cohorts.

### Implications – noise, screening, and intervention

Given the relationship between speech-in-noise recognition ability and literacy performance was identified only in the younger children in this study, further research should investigate whether this trend continues for younger children than those included in this research. Further research to explore the predictive value of determining speech-in-noise ability in younger children may help identify those at risk of developing reading and literacy delays. Assessing pre-school children would enable the introduction of not only early intervention strategies but may also indicate the appropriate teaching strategy for the individual. Seminal work from Castles and Rastle [64] has argued that a capacity for rapid abstraction of phonemic units and alphabetic decoding are necessary for successful early reading development. However, they also note a general resistance to explicit instruction in these areas due to a lack of theoretical knowledge of how these skills emerge and conflicting beliefs regarding instruction for their development. While current approaches to screening for PA deficits may provide some locus for increased focus on effective remediation approaches, screening for PA and decoding alone does not provide adequate insight into the underlying mechanism for the individuals’ learning delay. Successful training interventions for speech-in-noise ability deficits have also been shown [65], raising the question as to whether targeted remediation for speech-in-noise is targeting the PA component of listening and, if so, whether this training also transfers to improved literacy learning. While screening tools to flag speech-in-noise deficits exist (e.g., Sound Scouts), these are not routinely applied at school entry, suggesting we are missing a key opportunity for early identification of children at risk of developing a literacy deficit. Future research should also consider longitudinal studies to follow these associations in larger cohorts from a wider range of backgrounds spanning national educational programs. Consideration of long-term noise levels in classrooms and interactions of development of both literacy and listening in noise skills across time will also provide further insight.

## Conclusions

A significant interaction between speech-in-noise ability and literacy academic achievement was found, however this result was only seen in the younger cohort. This does not imply that literacy delays have resolved in older year levels. There is no significant difference seen between the variance of the NAPLAN results in each year level, indicating that the same proportion of children are evident in both cohorts who perform well below their peers. Whilst there are similar underlying processes involved in how children develop literacy skills and manage speech-in-noise, as children become older different factors such as stored knowledge, lived experience and cognitive skills will also start to influence performance on both reading and listening tasks. These factors, combined with the different developmental trajectories of the various underlying skills that contribute to literacy development and speech-in-noise recognition, results in the association between the two skills no longer being evident. Furthermore, the lack of any association between numeracy achievements and listening skills provides additional insight into the processes and skills required for literacy, numeracy and speech-in-noise recognition. In particular, the absence of association between numeracy and listening skills may support the important role phonological awareness plays in the development of listening ability. The present study highlights that the skills required to navigate noisy listening environments represent a significant impact on not only learning, but the ability of students to manage classroom listening environments. The impact of these skills on learning have implications for school screening initiatives, targeted teaching methodologies, and the importance of interventions aimed at improving the learning setting for every student.

## Supporting information

S1 File Dataset deidentified. (XLSX)

## References

[1] Khairi Md Daud M, Noor RM, Rahman NA, Sidek DS, Mohamad A. The effect of mild hearing loss on academic performance in primary school children. Int J Pediatr Otorhinolaryngol. 2010;74(1):67–70. doi: 10.1016/j.ijporl.2009.10.013 19913305

[2] Kuppler K, Lewis M, Evans AK. A review of unilateral hearing loss and academic performance: is it time to reassess traditional dogmata?. Int J Pediatr Otorhinolaryngol. 2013;77(5):617–22. doi: 10.1016/j.ijporl.2013.01.014 23474216

[3] Qi S, Mitchell RE. Large-scale academic achievement testing of deaf and hard-of-hearing students: past, present, and future. J Deaf Stud Deaf Educ. 2012;17(1):1–18. doi: 10.1093/deafed/enr028 21712463

[4] Spiegel JA, Goodrich JM, Morris BM, Osborne CM, Lonigan CJ. Relations between executive functions and academic outcomes in elementary school children: a meta-analysis. Psychol Bull. 2021;147(4):329–51. doi: 10.1037/bul0000322 34166004 PMC8238326

[5] McSporran E. Towards better listening and learning in the classroom. Edu Rev. 1997;49(1):13–20. doi: 10.1080/0013191970490102

[6] Rosenberg GG, Blake-Rahter P, Heavner J, Allen L, Redmond BM, Phillips J. Improving classroom acoustics (ICA): a three-year FM sound field classroom amplification study. J Educ Audiol. 1999;7:8–28.

[7] Crandell CC, Smaldino JJ. Classroom acoustics for children with normal hearing and with hearing impairment. Lang Speech Hear Serv Sch. 2000;31(4):362–70. doi: 10.1044/0161-1461.3104.362 27764475

[8] Nelson PB, Soli S. Acoustical barriers to learning: children at risk in every classroom. Lang Speech Hear Serv Sch. 2000;31(4):356–61. doi: 10.1044/0161-1461.3104.356 27764474

[9] Bigozzi L, Tarchi C, Vagnoli L, Valente E, Pinto G. Reading fluency as a predictor of school outcomes across grades 4-9. Front Psychol. 2017;8:200. doi: 10.3389/fpsyg.2017.00200 28261134 PMC5306315

[10] Klatte M, Bergström K, Lachmann T. Does noise affect learning? A short review on noise effects on cognitive performance in children. Front Psychol. 2013;4:578. doi: 10.3389/fpsyg.2013.00578 24009598 PMC3757288

[11] Yang W, Bradley JS. Effects of room acoustics on the intelligibility of speech in classrooms for young children. J Acoust Soc Am. 2009;125(2):922–33. doi: 10.1121/1.3058900 19206869

[12] Minelli G, Puglisi GE, Astolfi A. Acoustical parameters for learning in classroom: a review. Building Environ. 2022;208:108582. doi: 10.1016/j.buildenv.2021.108582

[13] Berg FS, Blair JC, Benson PV. Classroom acoustics. Lang Speech Hear Serv School. 1996;27(1):16–20. doi: 10.1044/0161-1461.2701.16

[14] Shield B, Greenland E, Dockrell J. Noise in open plan classrooms in primary schools: a review. Noise Health. 2010;12(49):225–34. doi: 10.4103/1463-1741.70501 20871177

[15] Puglisi GE, Prato A, Sacco T, Astolfi A. Influence of classroom acoustics on the reading speed: a case study on Italian second-graders. J Acoust Soc Am. 2018;144(2):EL144. doi: 10.1121/1.5051050 30180687

[16] Ellermeier W, Zimmer K. The psychoacoustics of the irrelevant sound effect. Acoust Sci Tech. 2014;35(1):10–6. doi: 10.1250/ast.35.10

[17] Tremblay S, Nicholls AP, Alford D, Jones DM. The irrelevant sound effect: does speech play a special role?. J Exp Psychol Learn Mem Cogn. 2000;26(6):1750–4. doi: 10.1037//0278-7393.26.6.1750 11185795

[18] Klatte M, Lachmann T, Schlittmeier S, Hellbrück J. The irrelevant sound effect in short-term memory: is there developmental change?. Eur J Cogn Psychol. 2010;22(8):1168–91. doi: 10.1080/09541440903378250

[19] Rance G, Dowell RC, Tomlin D. The effect of classroom environment on literacy development. NPJ Sci Learn. 2023;8(1):9. doi: 10.1038/s41539-023-00157-y 37012296 PMC10070343

[20] Shield BM, Dockrell JE. The effects of environmental and classroom noise on the academic attainments of primary school children. J Acoust Soc Am. 2008;123(1):133–44. doi: 10.1121/1.2812596 18177145

[21] Klatte M, Lachmann T, Meis M. Effects of noise and reverberation on speech perception and listening comprehension of children and adults in a classroom-like setting. Noise Health. 2010;12(49):270–82. doi: 10.4103/1463-1741.70506 20871182

[22] Mealings K. Classroom acoustics and cognition: a review of the effects of noise and reverberation on primary school children’s attention and memory. Building Acoustics. 2022;29(3):401–31. doi: 10.1177/1351010x221104892

[23] Mealings K. A scoping review of the effect of classroom acoustic conditions on primary school children’s numeracy performance and listening comprehension. Acoust Aust. 2022;51(1):129–58. doi: 10.1007/s40857-022-00284-3

[24] Klatte M, Hellbrück J, Seidel J, Leistner P. Effects of classroom acoustics on performance and well-being in elementary school children: a field study. Environ Behav. 2010;42(5):659–92. doi: 10.1177/0013916509336813

[25] Nittrouer S, Krieg LM, Lowenstein JH. Speech recognition in noise by children with and without dyslexia: how is it related to reading?. Res Dev Disabil. 2018;77:98–113. doi: 10.1016/j.ridd.2018.04.014 29724639 PMC5947872

[26] Van Hirtum T, Moncada-Torres A, Ghesquière P, Wouters J. Speech envelope enhancement instantaneously effaces atypical speech perception in dyslexia. Ear Hear. 2019;40(5):1242–52. doi: 10.1097/AUD.0000000000000706 30844835

[27] Australian Bureau of Statistics. Socio-Economic Indexes for Areas (SEIFA), Australia. 2023. https://www.abs.gov.au/statistics/people/people-and-communities/socio-economic-indexes-areas-seifa-australia/latest-release.

[28] Australian Curriculum Assessment and Reporting Authority (ACARA). NAPLAN 2023. https://acara.edu.au/assessment/naplan

[29] Dillon H, Mee C, Moreno JC, Seymour J. Hearing tests are just child’s play: the sound scouts game for children entering school. Int J Audiol. 2018;57(7):529–37. doi: 10.1080/14992027.2018.1463464 29703099

[30] Bowers P, Graydon K, Rance G. Evaluation of a game-based hearing screening program for identifying hearing loss in primary school-aged children. Int J Audiol. 2022:1–9. 10.1080/14992027.2022.2052981 35343856

[31] James G, Witten D, Hastie T, Tibshirani R. An introduction to statistical learning. Springer; 2013.

[32] Zuur AF, Ieno EN, Elphick CS. A protocol for data exploration to avoid common statistical problems. Methods Ecol Evol. 2010;1(1):3–14.

[33] Neter J, Wasserman W, Kutner MH. Applied linear regression models. Richard D. Irwin; 1983.

[34] Ching TY, Zhang VW, Flynn C, Burns L, Button L, Hou S, et al. Factors influencing speech perception in noise for 5-year-old children using hearing aids or cochlear implants. Int J Audiol. 2018;57(sup2):S70–80. doi: 10.1080/14992027.2017.1346307 28687057 PMC5756692

[35] Griffin AM, Poissant SF, Freyman RL. Speech-in-noise and quality-of-life measures in school-aged children with normal hearing and with unilateral hearing loss. Ear Hear. 2019;40(4):887–904. doi: 10.1097/AUD.0000000000000667 30418282 PMC7104694

[36] Porto L, Wouters J, van Wieringen A. Speech perception in noise, working memory, and attention in children: a scoping review. Hear Res. 2023;439:108883. doi: 10.1016/j.heares.2023.108883 37722287

[37] Thompson EC, Krizman J, White-Schwoch T, Nicol T, Estabrook R, Kraus N. Neurophysiological, linguistic, and cognitive predictors of children’s ability to perceive speech in noise. Dev Cogn Neurosci. 2019;39:100672. doi: 10.1016/j.dcn.2019.100672 31430627 PMC6886664

[38] McCreery RW, Spratford M, Kirby B, Brennan M. Individual differences in language and working memory affect children’s speech recognition in noise. Int J Audiol. 2017;56(5):306–15. doi: 10.1080/14992027.2016.1266703 27981855 PMC5634965

[39] Wagner RK, Torgesen JK. The nature of phonological processing and its causal role in the acquisition of reading skills. Psychol Bull. 1987;101(2):192.

[40] Werfel KL, Hendricks AE. The contribution of phonological processing to reading and spelling in students with cochlear implants. Lang Speech Hear Serv Sch. 2023;:1–14. 10.1044/2023_LSHSS-22-00129 37195296

[41] Duff F, Hulme C, Snowling M. Learning disorders and dyslexia. In: Friedman H, editor. Encyclopedia of mental health. 2nd edition. Oxford: Academic Press; 2016. p. 5–11.

[42] Grigorenko EL, Compton DL, Fuchs LS, Wagner RK, Willcutt EG, Fletcher JM. Understanding, educating, and supporting children with specific learning disabilities: 50 years of science and practice. Am Psychol. 2020;75(1):37–51. doi: 10.1037/amp0000452 31081650 PMC6851403

[43] Eccles R, VanderLinde J, leRoux M, Holloway J, MacCutcheon D, Ljung R. Is phonological awareness related to pitch, rhythm, and speech-in-noise discrimination in young children?. Lang Speech Hear Serv Sch. 2021;52(1):383–95. 33464981 10.1044/2020_LSHSS-20-00032

[44] Suggate SP. A meta-analysis of the long-term effects of phonemic awareness, phonics, fluency, and reading comprehension interventions. J Learn Disabil. 2016;49(1):77–96. doi: 10.1177/0022219414528540 24704662

[45] Goswami U, Huss M, Mead N, Fosker T. Auditory sensory processing and phonological development in high IQ and exceptional readers, typically developing readers, and children with dyslexia: a longitudinal study. Child Dev. 2021;92(3):1083–98. doi: 10.1111/cdev.13459 32851656

[46] Goswami U, Thomson J, Richardson U, Stainthorp R, Hughes D, Rosen S, et al. Amplitude envelope onsets and developmental dyslexia: a new hypothesis. Proc Natl Acad Sci U S A. 2002;99(16):10911–6. doi: 10.1073/pnas.122368599 12142463 PMC125072

[47] Corriveau KH, Goswami U, Thomson JM. Auditory processing and early literacy skills in a preschool and kindergarten population. J Learn Disabil. 2010;43(4):369–82. doi: 10.1177/0022219410369071 20457882

[48] Wimmer H, Landerl K, Linortner R, Hummer P. The relationship of phonemic awareness to reading acquisition: more consequence than precondition but still important. Cognition. 1991;40(3):219–49. doi: 10.1016/0010-0277(91)90026-z 1786676

[49] Boets B, Wouters J, van Wieringen A, Ghesquière P. Auditory processing, speech perception and phonological ability in pre-school children at high-risk for dyslexia: a longitudinal study of the auditory temporal processing theory. Neuropsychologia. 2007;45(8):1608–20. doi: 10.1016/j.neuropsychologia.2007.01.009 17303197

[50] Vandewalle E, Boets B, Ghesquière P, Zink I. Auditory processing and speech perception in children with specific language impairment: relations with oral language and literacy skills. Res Dev Disabil. 2012;33(2):635–44. doi: 10.1016/j.ridd.2011.11.005 22155538

[51] Ljung R, Sörqvist P, Hygge S. Effects of road traffic noise and irrelevant speech on children’s reading and mathematical performance. Noise Health. 2009;11(45):194–8. doi: 10.4103/1463-1741.56212 19805928

[52] Singer V, Strasser K, Cuadro A. Direct and indirect paths from linguistic skills to arithmetic school performance. J Educ Psychol. 2019;111(3):434.

[53] Amland T, Lervåg A, Melby-Lervåg M. Comorbidity between math and reading problems: is phonological processing a mutual factor?. Front Hum Neurosci. 2021;14:577304. 33488369 10.3389/fnhum.2020.577304 PMC7817538

[54] Vista A. The role of reading comprehension in maths achievement growth: investigating the magnitude and mechanism of the mediating effect on maths achievement in Australian classrooms. Int J Edu Res. 2013;62:21–35. doi: 10.1016/j.ijer.2013.06.009

[55] Serow P, Callingham R, Tout D. Assessment of mathematics learning: what are we doing? Research in mathematics education in Australasia 2012-2015. 2016. p.235-–54.

[56] Heidari A, Moossavi A, Yadegari F, Bakhshi E, Ahadi M. Effects of age on speech-in-noise identification: subjective ratings of hearing difficulties and encoding of fundamental frequency in older adults. J Audiol Otol. 2018;22(3):134–9. doi: 10.7874/jao.2017.00304 29719950 PMC6103490

[57] Gillon GT. Phonological awareness: from research to practice. Guilford Publications; 2017.

[58] Schmidt C, Brandenburg J, Busch J, Büttner G, Grube D, Mähler C, et al. Developmental trajectories of phonological information processing in upper elementary students with reading or spelling disabilities. Reading Res Quart. 2020;56(1):143–71. doi: 10.1002/rrq.299

[59] Dillon H, Cameron S. Separating the causes of listening difficulties in children. Ear Hear. 2021;42(5):1097–108. doi: 10.1097/AUD.0000000000001069 34241982 PMC8378540

[60] Fallon M, Trehub SE, Schneider BA. Children’s use of semantic cues in degraded listening environments. J Acoust Soc Am. 2002;111(5 Pt 1):2242–9. doi: 10.1121/1.1466873 12051444

[61] Tomlin D, Dillon H, Sharma M, Rance G. The impact of auditory processing and cognitive abilities in children. Ear Hear. 2015;36(5):527–42. 25951047 10.1097/AUD.0000000000000172

[62] Gale A, Lynn R. A developmental study of attention. Br J Educ Psychol. 1972;42(3):260–6. doi: 10.1111/j.2044-8279.1972.tb00719.x 4643755

[63] Rebok GW, Smith CB, Pascualvaca DM, Mirsky AF, Anthony BJ, Kellam SG. Developmental changes in attentional performance in urban children from eight to thirteen years. Child Neuropsychology. 1997;3(1):28–46. doi: 10.1080/09297049708401366

[64] Castles A, Rastle K, Nation K. Ending the reading wars: reading acquisition from novice to expert. Psychol Sci Public Interest. 2018;19(1):5–51. doi: 10.1177/1529100618772271 29890888

[65] Cameron S, Dillon H. Development and evaluation of the LiSN & learn auditory training software for deficit-specific remediation of binaural processing deficits in children: preliminary findings. J Am Acad Audiol. 2011;22(10):678–96. doi: 10.3766/jaaa.22.10.6 22212767

